# Atorvastatin combined with poly-unsaturated fatty acid confers better improvement of dyslipidemia and endothelium function

**DOI:** 10.1186/1476-511X-13-186

**Published:** 2014-12-10

**Authors:** Xianbing Song, Hongsheng Liu, Xiaotian Wang, Zhenhua Li, Congwu Huang

**Affiliations:** Department of Human anatomy, Anhui Medical College, Hefei, 230601 China; Emergency Department of the First Affiliated Hospital of Chinese PLA General Hospital, Beijing, 10048 China; Department of Cardiology, Dongying People’s Hospital, Shandong, 257000 China; Department of Internal Medicine, the Second Affiliated Hospital of Shantou University, Guangdong, 51000 China

**Keywords:** Dyslipidemia, Endothelium function, Synergistic effect

## Abstract

**Background:**

Atorvastatin and poly-unsaturated fatty acid (PUFA) are beneficial for lipid-modification, whether atorvastatin plus PUFA could confer better improvement on dyslipidemia and endothelium function is unknown.

**Methods:**

Dyslipidemia model of 40 rabbits were produced with atherogenic diet, and thereafter saline, atorvastatin, PUFA, or atorvastatin plus PUFA were prescribed for 1 week. Ten rabbits given normal diet served as the sham group. Parameters of interest including lipid profiles, endothelium function (nitric oxide, NO) and activation (solution vascular-cellular adhesion molecule, (sVCAM) and intracellular adhesion molecule, (sICAM)), markers of inflammation (C-reactive protein, CRP) and oxidation (malondialdehyde, MDA) were compared among groups.

**Results:**

There was no significant difference of parameters among groups at the initial. With 1 week of atherogenic diet administration, serum levels of lipid profiles, sVCAM and sICAM, CRP and MDA were significantly increased, accompanying with profound NO reduction, as compared to the sham group. After 1 week of medical intervention, as compared to the control group (saline administration), dyslipidemia and endothelium function were modestly improved with either atorvastatin or PUFA therapy. Nevertheless, these efficacies were further and significantly enhanced with combined therapy when compared to the control group (p < 0.005), suggesting that there was synergistic effects of atorvastatin and PUFA co-therapy in rabbits with dyslipidemia.

**Conclusion:**

Atorvastatin plus PUFA therapy could immediately contribute to better improvement of lipid-modification and endothelium function in rabbits with dyslipidemia.

## Introduction

Dyslipidemia, predominantly characterized by elevated serum levels of low density lipoprotein-cholesterol (LDL-C) and triglyceride and decreased high density lipoprotein-cholesterol (HDL-C), is the major cause of atherosclerosis and atherosclerotic cardiovascular diseases [1, 2]. 3-hydroxy-3-methylglutaryl coenzyme-A reductase (HMG-CoA reductase) inhibitor, statin, has been broadly applied to treat dyslipidemia and atherosclerosis [3, 4]. Other than statin, in the past decades, some experimental studies showed that poly-unsaturated fatty acid (PUFA) were also beneficial for lipid-modification [5, 6]. Additionally, data from some epidemiological studies indicated that increased PUFA consumption could not only result in better improvement of dyslipidemia, but also be possible to retard the progression of atherosclerosis [7–9]. Nevertheless, since nearly almost every patient with dyslipidemia is treated with statin currently, therefore whether the benefit of PUFA render to lipid-modification is independent of or is overlapped with statin therapy is deserved to be further investigated, especially its associated mechanisms. Additionally, whether statin combined with PUFA could better improve dyslipidemia and endothelium function in the early stage is also unclear.

In light of previous findings, we conducted an experimental study to compare the efficacy of statin and PUFA on lipid-modification in rabbits with dyslipidemia. Moreover, since endothelium dysfunction and activation is implicated in the early stage of atherosclerosis under the setting of dyslipidemia, the effect of PUFA on endothelial function and its associated mechanisms would also be evaluated herein. We believed that data from our current study would provide more evidence regarding the effect and application of PUFA on the primary and secondary prevention of atherosclerotic cardiovascular diseases in subjects with dyslipidemia.

## Methods

### Dyslipidemia animal model production

Totally 50 male New Zealand White rabbits, weighing 1.2-1.5 kilogram and with 6–8 weeks old, were obtained from Experimental Animal Center of Shantou University, and the protocol of current study was approved by Ethic Committee of Shantou University. Briefly, 10 rabbits were served as the sham group, and another 40 rabbits were used as dyslipidemia group, which were prescribed with atherogenic diet containing 0.5% cholesterol for 1 week as described by previous study [10].

### Studied protocol

Rabbits with dyslipidemia were randomly and evenly assigned into the control group (3 ml of normal saline administration), atorvastatin group (5 mg/kg body weight/day of atorvastatin reconstituted in 3 ml normal saline, oral administration), PUFA group (Eicosapentaenoic acid/Docosahexaenoic acid, EPA/DHA with 1:1 ratio, 10 ml/day was added into diet), and combined group (prescribed the same doses of atorvastatin and PUFA as above), and the sham group was prescribed with 3 ml normal saline. The duration of intervention lasted for 1 week.

### Laboratory examination

At initial, 1 week of atherogenic diet administration, and 1 week after medical intervention, fasting blood was sampled for assessing parameters of interest, including lipid profile (including TG, TC, LDL-C and HDL-C), nitric oxide (NO) production, serum levels of C-reactive protein (CRP), solution vascular-cellular adhesion molecule (sVCAM), solution intracellular adhesion molecule (sICAM) and malondialdehyde (MDA), were evaluated. All procedures were performed according to the manufacture instruction and three independent experiments were performed in duplicate.

### Statistical analyses

Continuous variable was presented as mean ± SD and compared by the Student’s t-test when data was normally distributed, otherwise compared by Wilcoxon rank-sum test. Statistical analyses were performed by using SPSS software version 18.0 (SPSS, Inc., Chicago, Illinois). A value of p <0.05 was considered significant.

## Results

### Comparison of parameters between the sham and dyslipidemia groups

Briefly, parameters of interest at the initial and at 1 week of atherogenic diet treatment were compared among groups. As presented in Table 1, there was no significant difference of lipid profile, NO production, serum levels of CRP, sVCAM, sICAM and MDA between the sham and dyslipidemia groups. Nevertheless, with 1 week of atherogenic diet administration, except HDL-C, serum levels of TG, TC, LDL-C were significantly increased in the dyslipidemia group as compared to the sham group (p < 0.05). Additionally, NO production was profoundly declined in accompany with elevation of CRP, sVCAM, sICAM and MDA in the dyslipidemia groups (p < 0.05).

**Table 1:** **Parameters comparison between the sham and dyslipidemia groups**

Variables	Sham (n = 10)	Control (n = 10)	Atorvastatin (n = 10)	PUFA (n = 10)	Combined (n = 10)
**At initial**					
TG (mmol/L)	1.08 ± 0.11	1.03 ± 0.12	1.04 ± 0.10	1.06 ± 0.11	1.04 ± 0.12
TC (mmol/L)	3.82 ± 0.21	3.80 ± 0.16	3.81 ± 0.17	3.78 ± 0.16	3.78 ± 0.14
LDL-C (mmol/L)	2.03 ± 0.15	1.98 ± 0.12	2.01 ± 0.12	2.00 ± 0.12	2.01 ± 0.13
HDL-C (mmol/L)	0.92 ± 0.06	0.92 ± 0.05	0.93 ± 0.07	0.91 ± 0.03	0.90 ± 0.07
NO (μmol/L)	12.24 ± 1.23	12.09 ± 1.16	12.13 ± 1.17	12.20 ± 1.09	12.12 ± 1.18
CRP (mg/L)	2.08 ± 0.36	2.11 ± 0.23	2.04 ± 0.29	2.10 ± 0.19	2.11 ± 0.24
sVCAM (μg/mL)	3.52 ± 0.26	3.57 ± 0.33	3.49 ± 0.20	3.50 ± 0.30	3.52 ± 0.35
sICAM (μg/mL)	0.43 ± 0.06	0.41 ± 0.03	0.44 ± 0.05	0.43 ± 0.05	0.42 ± 0.05
MDA (nmol/L)	0.86 ± 0.05	0.89 ± 0.05	0.82 ± 0.04	0.86 ± 0.07	0.85 ± 0.04
**1 week of atherogenic diet treatment**					
TG (mmol/L)	1.10 ± 0.12*	1.23 ± 0.18	1.24 ± 0.13	1.20 ± 0.09	1.22 ± 0.06
TC (mmol/L)	3.85 ± 0.20*	4.35 ± 0.13	4.40 ± 0.17	4.41 ± 0.12	4.38 ± 0.11
LDL-C (mmol/L)	2.06 ± 0.12*	2.35 ± 0.26	2.32 ± 0.32	2.30 ± 0.26	2.30 ± 0.21
HDL-C (mmol/L)	0.93 ± 0.06	0.90 ± 0.06	0.90 ± 0.07	0.91 ± 0.03	0.92 ± 0.05
NO (μmol/L)	12.18 ± 1.09*	9.26 ± 1.02	9.23 ± 1.06	9.25 ± 1.02	9.20 ± 1.07
CRP (mg/L)	2.07 ± 0.32*	4.63 ± 0.43	4.61 ± 0.34	4.58 ± 0.30	4.60 ± 0.26
sVCAM (μg/mL)	3.50 ± 0.25*	4.95 ± 0.42	4.92 ± 0.36	4.92 ± 0.18	4.93 ± 0.27
sICAM (μg/mL)	0.44 ± 0.05*	0.58 ± 0.07	0.56 ± 0.06	0.63 ± 0.08	0.65 ± 0.06
MDA (nmol/L)	0.88 ± 0.04*	1.09 ± 0.06	1.05 ± 0.04	1.07 ± 0.04	1.07 ± 0.06

Denote: *p < 0.05 versus other groups.

### Comparison of effects of different interventions

With 1 week’s medical intervention, parameters of interest among groups were evaluated so as to compare the effects of different interventions. As shown in Table 2, in comparison to the control group, serum levels of TG, TC and LDL-C were slightly but not significantly reduced in the atorvastatin or PUFA therapy groups (p > 0.05). The modest improvement of NO production, and marginal reduction of serum levels of CRP, sVCAM, sICAM and MDA were also observed between the control and the atorvastatin and PUFA therapy groups (p > 0.05). Nevertheless, in comparison to the control group, the improvements of lipid profiles, endothelium dysfunction and activation and systemic inflammation and oxidation were profoundly enhanced by combined therapy (p < 0.05), strongly suggesting that atorvastatin plus PUFA administration had synergistic protective effects in rabbits with dyslipidemia.

**Table 2:** **Comparison of effects of different intervention**

Variables	Sham (n = 10)	Control (n = 10)	Atorvastatin (n = 10)	PUFA (n = 10)	Combined (n = 10)
TG (mmol/L)	1.07 ± 0.09	1.26 ± 0.14	1.18 ± 0.12	1.20 ± 0.17	1.11 ± 0.08^#^
TC (mmol/L)	3.84 ± 0.15	4.32 ± 0.11	4.16 ± 0.16	4.18 ± 0.13	4.06 ± 0.06^#^
LDL-C (mmol/L)	2.02 ± 0.11	2.33 ± 0.20	2.17 ± 0.25	2.19 ± 0.17	2.10 ± 0.12^#^
HDL-C (mmol/L)	0.92 ± 0.03	0.91 ± 0.03	0.92 ± 0.04	0.95 ± 0.06	1.01 ± 0.03^#^
NO (μmol/L)	12.18 ± 1.16	9.02 ± 1.00	10.27 ± 1.14	10.14 ± 1.08	10.88 ± 1.04^#^
CRP (mg/L)	2.10 ± 0.39	4.68 ± 0.37	3.67 ± 0.42	3.72 ± 0.33	3.28 ± 0.30^#^
sVCAM (μg/mL)	3.48 ± 0.22	4.98 ± 0.24	4.46 ± 0.53	4.49 ± 0.27	4.17 ± 0.38^#^
sICAM (μg/mL)	0.40 ± 0.04	0.58 ± 0.05	0.53 ± 0.03	0.53 ± 0.04	0.49 ± 0.04^#^
MDA (nmol/L)	0.82 ± 0.06	1.10 ± 0.08	1.02 ± 0.03	1.03 ± 0.03	0.96 ± 0.05^#^

Denote: ^#^p < 0.05 versus control group.

## Discussion

With regard to its potent lipid-lowering and pleiotropic effects, statin has been defined as the cornerstone of dyslipidemia and atherosclerosis therapy [3, 4]. Poly-unsaturated fatty acid, a significant and essential nutrition, now has also been recommended by some expertise of cardiology and nutrition for the adjunctive therapy of dyslipidemia and atherosclerotic cardiovascular diseases [11–13]. But the mechanisms of PUFA on dyslipidemia-modification and atherosclerosis-prevention are far less clear. Moreover, whether statin plus PUFA therapy could confer more robust efficacy on cardiovascular system needs further investigation. Data from our current experimental study show that in rabbits with dyslipidemia, as compared to the control group, 1 week of atorvastatin or PUFA therapy slightly improve dyslipidemia. Moreover, markers of endothelium dysfunction and activation as well as inflammation and oxidative stress are also modestly ameliorated. When atorvastatin combined with PUFA administration, however, the efficacies are further enhanced as compared to either atorvastatin or PUFA therapy alone, strongly suggesting synergistic effects exit between statin and PUFA in treatment of early stage of dyslipidemia.

Basically, disorders of lipid metabolism not only results from over-consumption of cholesterol and triglyceride, but also associated with the alteration of the profile of individual lipid molecule [14, 15]. Via inhibiting HMG-CoA reductase, statin blocks the process of cholesterol synthesis. Additionally, by means of reducing the generation of farnesylpyrophosphate (FPP) and geranyl-geranylpyrophosphate (GGPP), statin exerts its pleiotropic effects such as improving endothelium function, ameliorating inflammation and oxidation, promoting angiogenesis and anti-apoptosis [16, 17]. Accordingly, the early stage of endothelium dysfunction and activation majorly manifested as decreased NO production and increased sICAM and sVCAM generation [18, 19]. Data from our current study was consistent with previous findings [19, 20]. As compared to the control group, parameters of interest including lipid profile and endothelium function were slightly improved in the atorvastatin group, although without significant difference. To our best knowledge, it might be due to either short-term or low-dose of atorvastatin administration. Previously [21], our study showed that in rats with dyslipidemia, 10 mg/kg body weight/day of atorvastatin administration for 2 weeks significantly improved dyslipidemia as compared to the control group. In addition, serum levels of CRP and MDA were also modestly reduced. Of note, since our current study was to evaluate whether PUFA could exert protective effect on endothelium function and whether PUFA could improve dyslipidemia immediately, therefore we pre-defined 1 week of intervention as the time-point for outcome assessment. In light of previous reports and our published study, we believed that it was doubtless that atorvastatin could profoundly correct the disorder of lipid metabolism and endothelium dysfunction with time.

Notably, eicosapentaenoic acid (EPA) and docosahexenoic acid (DHA) are the two key components of PUFA and there were studies showing that EPA and DHA were beneficial for lipid-modification, hypertension-management and glycemic-control [22, 23]. Moreover, increased PUFA consumption may retard the progression of atherosclerosis and even reduce the incidence of cardiovascular events [22–25]. Consistent with previous studies [26, 27], data from our study also revealed that 10 ml/day of EPA/DHA with 1:1 ratio marginally improved dyslipidemia and endothelium function, although without statistical significance. We speculated that it might be also related with the short-term use of PUFA. In the future, it is imperative to address whether prolonged PUFA administration could result in better improvement of dyslipidemia and endothelium function. Importantly, contrast to the slight improvement with either atorvastatin or PUFA treatment, atorvastatin combined with PUFA profoundly improved both dyslipidemia and endothelium function, even with 1 week’s administration, suggesting that there might be robust synergistic effects of atorvastatin and PUFA on treating early stage of dyslipidemia and endothelium dysfunction. To our best knowledge, some mechanisms might be attributed to this finding. In the first place, both statin and PUFA are capable to modify dyslipidemia as reflected in previous reports and our current study [21, 28]. However, when used alone with a short duration, these benefits of statin or PUFA therapy might not possible turn into prominence. Therefore, co-therapy with statin and PUFA might enhance the modest efficacy of lipid-modification and endothelium-improvement immediately. Secondly, as mentioned before that statin could inhibit cholesterol biosynthesis while PUFA could improve the disorder of plasma lipid composition. And data from our study also revealed that with combined therapy, not only TG, TC and LDL-C were reduced but also HDL-C was increased as compared to the control group. As is well known that increased HDL-C level is beneficial for retarding atherosclerosis progression [29]. Therefore, we considered that statin combined with PUFA therapy might through different and complement mechanisms to result in better improvement of dyslipidemia and endothelium dysfunction. Last but not the least, both statin and PUFA have benefits beyond lipid-modification as aforementioned above, therefore when used in combination, these pleiotropic protective effects might synergy and contribute to better improvement of lipid disorder and endothelium dysfunction.

## Conclusion

In conclusion, atorvastatin plus PUFA therapy could immediately contribute to better improvement of lipid disorder and endothelium dysfunction in rabbits with dyslipidemia, and future study is warranted to investigate whether atorvastatin combined with PUFA could robustly retard established atherosclerotic plaques.

## Authors’ information

Xianbing Song and Hongsheng Liu co-first authors.
